# The cross-sectional relationship between pain and awareness of age-related changes

**DOI:** 10.1177/2049463720961798

**Published:** 2020-10-01

**Authors:** Serena Sabatini, Obioha C Ukoumunne, Clive Ballard, Rachel Collins, Anne Corbett, Helen Brooker, Linda Clare

**Affiliations:** 1 School of Psychology, University of Exeter, Exeter, UK; 2 College of Medicine and Health, University of Exeter, Exeter, UK; 3 NIHR ARC South West Peninsula (PenARC), University of Exeter, Exeter, UK; 4 Ecog Pro Ltd, Bristol, UK

**Keywords:** AARC, self-perceptions of health, healthy ageing, older individuals, subjective ageing

## Abstract

**Background:**

Awareness of positive and negative age-related changes (AARC gains and losses) captures the perceived changes that older individuals experience in several domains of their lives including physical, cognitive and social functioning; interpersonal relationships; and lifestyle. Exploring antecedents of AARC is important to identify those individuals that could benefit the most from interventions promoting positive experiences of ageing and/or adaptation to age-related changes. This study investigates the experience of pain as a predictor of lower AARC gains and higher AARC losses.

**Methods:**

Analyses are based on cross-sectional data from the PROTECT cohort (2019); 1013 UK residents (mean (SD; range) age: 65.3 (7.1; 51.4–92) years, 84.4% women) completed measures of AARC and pain and provided demographic information. Linear regression models were fitted to examine pain as a predictor of AARC gains and AARC losses.

**Results:**

Higher levels of pain predicted more AARC losses both before (regression coefficient, *B* = 0.36; 95% confidence interval (CI): 0.29 to 0.42, *p*-value < 0.001; *R*^2^ = 0.11) and after adjusting for demographic covariates (*B* = 0.34; 95% CI: 0.27 to 0.40; *p*-value < 0.001; Partial *R*^2^ = 0.11). Pain was not significantly associated with AARC gains (unadjusted *B* = 0.05; 95% CI: −0.03 to 0.12, *p*-value = 0.21; Partial *R*^2^ = 0.01).

**Conclusion:**

Individuals experiencing pain may perceive more AARC losses. Interventions aiming to decrease levels of pain could include a component targeting self-perceptions of ageing and/or promoting acceptance of the negative changes that can happen with ageing.

**Statement of significance:**

The predictive role of greater levels of pain for more negative perceptions of age-related changes extends the literature on the negative psychological outcomes of pain and on predictors of perceived awareness of age-related changes (AARC). As individuals experiencing pain may be more at risk of perceiving their own ageing in a more negative way, they may benefit from interventions that combine strategies to reduce levels of pain and the interference that pain exerts on their daily activities with an educational component enhancing positive self-perceptions of ageing and promoting acceptance of negative age-related changes.

## Background

The proportion of older individuals in the world is increasing.^1^ As older age is a risk factor for poor physical health,^2^ the global number of people experiencing poor physical health is also increasing.^3^ The prevalence of chronic conditions in the United Kingdom has been estimated at 36% in individuals aged 65–74 and 47% in those aged 75 and over.^4^ The prevalence of multi-morbidities amounts to 46% among individuals aged 65–74 and to 69% among those aged 85 and over.^5^ As a consequence, many older individuals have to deal with different aspects of their physical health including taking medications and pain control. For instance, chronic pain affects between 35% and 51% of the UK adult population.^6^ The wide number of people experiencing poor health may be one of the reasons why many people perceive older age in a negative way.^7^ Indeed existing research suggests that individuals with poorer current and/or past physical health report more negative experiences of ageing.^8,9^ However, so far studies have explored the predictive role of physical health on bimodal measures of perceptions of ageing; using this approach, a person can report either positive or negative self-perceptions of ageing, but not both. The concept of awareness of age-related changes (AARC^10^) instead makes it possible to separately explore whether physical health explains variability in individuals’ perceived awareness of positive age-related changes (AARC gains) and/or negative age-related changes (AARC losses). AARC captures individuals’ perceptions that with increasing age, their behaviour, level of performance or way of experiencing life has changed. AARC assumes that people experience both positive (AARC gains) and negative (AARC losses) age-related changes. Moreover, individuals’ perceptions of changes can vary across five life domains: health and physical functioning, cognitive functioning, interpersonal relationships, socio-cognitive and socio-emotional functioning, and lifestyle/engagement.^10^ Perceived AARC gains are fairly independent from perceived AARC losses; indeed correlations between perceived gains and losses are small and positive.^11^

The concept of AARC shows stronger associations with perceived health^11,12^ compared to similar concepts such as subjective age and attitudes towards own ageing. This study focuses on an indicator of health that so far has not been explored in association with perceived AARC: pain. During qualitative analyses of individuals’ thoughts in relation to their perceived AARC, we found that some individuals attributed changes in their perceived levels of AARC to the experience of pain (unpublished data, available from the first author on request). It may be that the limitations related to pain foster or heighten perceptions of AARC losses.^10,13^ A recent study found that pain predicts more negative attitudes towards ageing among older people.^14^ The presence of pain can indeed restrict individuals’ independence, social engagement, contribution to society and emotional well-being and accelerate cognitive decline.^15,16^ These changes, often associated with the experience of pain, may then lead to higher perceived awareness of negative age-related changes. This study uses a large sample of UK individuals aged 50 and over to test whether higher pain is associated with lower levels of perceived gains and higher levels of perceived losses in the five AARC life domains. Understanding predictors of perceived AARC gains and losses is important in order to identify which groups of individuals may benefit the most from interventions aiming to promote more positive experiences of ageing^17^ and/or acceptance of negative age-related changes.^18^

## Study design and participants

This study is based on analyses of cross-sectional data collected in 2019 as part of the ongoing ‘PROTECT’^19^ study. ‘PROTECT’ is a longitudinal study launched in 2014 that assesses participants every year on measures of physical, mental and cognitive health; lifestyle; and perceptions of ageing through an online platform. Participants complete the measures online through the ‘PROTECT’ platform. Individuals are eligible to participate in the ‘PROTECT’ study if they are UK residents, English speakers, aged 50 years and over, have access to a computer and Internet, and do not have a clinical diagnosis of dementia at the point of recruitment. In this study, we analysed data only for those participants who completed measures of both pain and AARC between January and March 2019.

To recruit participants, leaflets containing information about the ‘PROTECT’ study were placed in GP surgeries and memory clinics within the United Kingdom. The study was also publicised through communication channels at King’s College London (KCL), the Biomedical Research Centre at KCL, the University of Exeter and the Royal Devon and Exeter NHS Trust; including use of the media, University press partners and online content. In addition, participants from existing cohort studies and/or online portals supporting recruitment^20–22^ were invited to take part in the ‘PROTECT’ study. Potential participants enrolled through the ‘PROTECT’ study website and provided consent online. The ‘PROTECT’ study has ethical approval from the London Bridge NHS Research Ethics Committee and Health Research Authority (Ref: 13/LO/1578). Ethical approval for the data analysis was sought through the ethics committee at the the University of Exeter, School of Psychology (Application ID: eCLESPsy000603 v1.0).

## Measures

### Pain

Perceived pain was assessed with a 5-item Pain Scale (Adapted from the Patient-Reported Outcomes Measurement Information System (PROMIS) pain scale).^23^ Respondents were asked to report how much pain has interfered with their daily activities and social engagement over the past 7 days. An example item is ‘In the past 7 days how much did pain interfere with your household chores?’. Participants answer each item on a scale from 0 to 4 (0 = *not at all*; 4 = *very much*). A total score is obtained by summing single-item scores. The total score ranges from 0 to 20; higher scores indicate greater levels of pain. In this study sample, Cronbach’s alpha (α) for the 5-item Pain Scale is 0.97.

### AARC

We assessed perceived age-related gains (AARC gains) and losses (AARC losses) with the AARC-10 SF.^11^ The AARC-10 SF contains 10 items, 5 assessing AARC gains and 5 assessing AARC losses. Each of these five items assesses a different AARC behavioural domain (health and physical functioning, cognitive functioning, interpersonal relationships, socio-cognitive and socio-emotional functioning, and lifestyle/engagement). Items included in the AARC-10 SF are reported in Table 1. Respondents rate how much each item applies to them on a 5-point Likert-type scale (from 1 = ‘not at all’ to 5 = ‘very much’). Scores for the AARC gains and AARC losses subscales are obtained by summing items that fall into the respective scales. Scale scores range from a possible 5 to 25 with higher scores indicating higher levels of AARC. In the UK validation of the AARC-10 SF, Cronbach’s alpha (α) value was 0.77 for the AARC gains subscale and 0.80 for the AARC losses subscale^24^ and therefore shown to be internally consistent for the UK population. This study is based on a subsample of participants taken from the broader sample of participants in the UK validation of the AARC-10 SF. In the current sample, Cronbach’s alpha (α) is 0.75 for the AARC gains subscale and 0.80 for the AARC losses subscale.

**Table 1.** AARC-10 SF items.

AARC-10 SF items		
With my increasing age, I realise that . . .		
Scale	AARC domain	Items
AARC-GAINS	PHYS	. . . I pay more attention to my health
	COG	. . . I have more experience and knowledge to evaluate things and people
	INT	. . . I appreciate relationship and people much more
	SCSE	. . . I have a better sense of what is important for me
	LIFE	. . . I have more freedom to live my days the way I want
AARC-LOSSES	PHYS	. . . I have less energy
	COG	. . . my mental capacity is declining
	INT	. . . I feel more dependent on the help of others
	SCSE	. . . I find it harder to motivate myself
	LIFE	. . . I have to limit my activities

AARC: awareness of age-related change; PHY: health and physical functioning; COG: cognitive functioning; INT: interpersonal relations; SCSE: social-cognitive and social-emotional functioning; LIFE: lifestyle and engagement.

## Variables used to describe the study sample

### Demographic variables

Participants provided demographic information through the ‘PROTECT’ online platform on age, sex, ethnic origin, marital status, education and employment status. Some demographic variables (age, sex, education and employment status) are used as covariates in this study.

### Indicators of mental health

We used the Patient Health Questionnaire-9 (PHQ-9)^25^ to assess the frequency of depressive symptoms over the previous 2 weeks. The PHQ-9 includes nine items, each using a 1 to 4 rating scale. The total score is the sum of single items scores and can range from a possible 9 to 36, with higher scores indicating greater experience of depressive symptoms. In this study sample, Cronbach’s alpha (α) for the PHQ-9 is 0.80.

We used the Generalized Anxiety Disorder-7 scale (GAD-7)^26^ to assess the frequency of symptoms of anxiety over the past 2 weeks. The GAD-7 includes seven items each rated on a scale from 1 to 4. The overall score is the sum of the items scores and ranges from a possible 7 to 28. Higher scores indicate greater presence of anxiety symptoms. In this study sample, Cronbach’s alpha (α) for the GAD-7 is 0.89. We assessed depressive and anxiety symptoms to describe the average levels of depression and anxiety in the study sample.

### Indicators of physical health

We used Lawton’s Instrumental Activities of Daily Living (IADL) scale^27^ to assess everyday functional status. The IADL describes seven activities, each rated from 0 to 2, including preparing meals, managing medications and using the telephone. The total score ranges from a possible 0 to 14; higher scores indicate greater difficulty in performing daily activities. In this study sample, Cronbach’s alpha (α) for the IADL scale is 0.81.

To assess perceived health, we used a single-item question(taken from the SF-36)^28^ asking participants to rate their own health on a 4-point scale ranging from excellent to poor (‘excellent’, ‘good’, ‘fair’, and ‘poor’). We assessed indicators of physical health to describe the functional status and health status of the study sample.

### Analyses

We estimated correlations between pain and perceived AARC gains and losses. Generally, correlation coefficients ⩽ 0.09 are considered negligible, between 0.10 and 0.29 are considered small, between 0.30 and 0.49 are considered moderate and ⩾0.50 are considered large.^29^ We fitted linear regression models to test our hypotheses that higher levels of perceived pain predict lower levels of perceived AARC gains and higher levels of perceived AARC losses. For each of the gains and losses outcomes, we first examined pain as the sole predictor (unadjusted model) and then in a second model we added potential confounding covariates (adjusted model) that were identified from previous literature on AARC.^30–33^ The covariates were participants’ age, gender, education level and current employment status. We checked that our data met the assumptions of the model (normality of residuals, linearity and homogeneity of variance).

## Results

### Study sample

Between 1 January 2019 and 31 March 2019, 1013 participants enrolled in the ‘PROTECT’ study answered both the pain questionnaire and the AARC-10 SF. Participants’ ages ranged from 51 to 92 years (*M* age = 65.3 years, SD = 7.1). The majority of participants were women (84.4%), White, unemployed, married and well-educated. On average, participants reported ‘quite a lot’ of AARC gains (*M* (SD) = 18.7 (3.7)) and ‘quite a bit’ of AARC losses (*M* (SD) = 10.1 (3.4)). The majority of participants did not report pain (78.0%); 12.6% of participants reported ‘a little bit’ of pain, 5.7% of participants reported experiencing ‘somewhat’ pain, only 2.8% of participants reported ‘quite a lot’ of pain and 0.9% reported experiencing ‘very much’ pain. The majority of participants perceived their health as being good (54.7%) or excellent (28.8%) and had no difficulties in conducting activities of daily living. On average, participants reported mild symptoms of depression (*M* (SD) = 11.5 (3.2)) and anxiety (*M* (SD) = 8.6 (2.9)). Further demographic characteristics of the study sample are reported in Table 2.

**Table 2.** Descriptive statistics of demographic and main study variables (N = 1013).

Age in years, mean (SD; range)	65.3 (7.1; 51.4–92.0)
Gender (women %)	84.4
Ethnicity (%)	
White	97.9
Mixed	0.4
Asian	1.2
Black	0.0
Other ethnic groups	0.5
Marital status (%)	
Married/civil partnership/co-habiting	78.8
Widowed/separated/divorced/single	21.2
Education level (%)	
Secondary	14.1
Post-secondary	9.6
Vocational qualification	19.7
Undergraduate degree	34.0
Post-graduate degree	19.2
Doctorate	3.4
Current employment (Yes %)	39.5
AARC gains, mean (SD)	18.7 (3.7)
AARC losses, mean (SD)	10.1 (3.4)
Pain (%)	
Not at all	78.0
A little bit	12.6
Somewhat	5.7
Quite a lot	2.8
Very much	0.9
Perceived health (%)	
Poor	2.8
Fair	13.7
Good	54.7
Excellent	28.8
IADL, mean (SD)	0.2 (1.0)
Depressive symptoms, mean (SD)	11.5 (3.2)
Anxiety symptoms, mean (SD)	8.6 (2.9)

AARC: awareness of age-related change; IADL: Instrumental Activities of Daily Living; SD: standard deviation; GAD-7: Generalized Anxiety Disorder-7; PROMIS: Patient-Reported Outcomes Measurement Information System.

Ethnic group was operationalised as a categorical variable including the following ethnicities: White, mixed, Asian, Black or other ethnic groups. Marital status was operationalised as a dichotomous variable capturing whether the participant is married, in a civil partnership, co-habiting versus widowed, separated, divorced or single. Education level was operationalised as a categorical variable including the following options: Secondary education, post-secondary education, vocational qualification, undergraduate degree, post-graduate degree and doctorate. Secondary Education = GCSE/O-Levels. Post-Secondary Education = College, A-Levels, NVQ3 or below, or similar. Vocational qualification = Diploma, Certificate, BTEC, NVQ 4 and above, or similar. Undergraduate Degree = BA, BSc or similar. Post-graduate Degree = MA, MSc or similar. Doctorate = PhD. Employment status was operationalised as a dichotomous variable capturing whether the participant is working or not. AARC-10 SF gains = scale assessing AARC gains in the five AARC life domains. AARC-10 SF losses = scale assessing AARC losses in the five AARC life domains. Pain = Participant’s score on the PROMIS pain scale. Perceived health = participants rated their own health on a 4-point scale ranging from excellent to poor ‘excellent’, ‘good’, ‘fair’ and ‘poor’. IADL = Instrumental Activities of Daily Living. Depressive symptoms = participants’ score on the Patient Health Questionnaire-9. Anxiety symptoms = participants’ score on the GAD-7.

### Correlations among study variables

Pain was positively but not significantly correlated with perceived AARC gains (*r* = 0.04; 95% confidence interval (CI): −0.22 to 0.10; *p*-value = 0.21). Pain was positively and significantly correlated with perceived AARC losses (*r* = 0.33; 95% CI: 0.28 to 0.39; *p*-value < 0.001). This means that those individuals who reported higher levels of pain also reported experiencing higher levels of perceived AARC losses. Correlation coefficients for perceived AARC gains and AARC losses with demographic variables are reported in Table 3.

**Table 3.** Pearson’s *r* correlations among study variables.

	AARC gains			AARC losses		
	Pearson’s *r*	95% CI	*p-*value	Pearson’s *r*	95% CI	*p-*value
Pain	0.04	−0.2 to 0.10	0.21	0.33	0.28 to 0.39	<0.0001
Age	−0.03	−0.09 to 0.03	0.34	0.20	0.14 to 0.26	<0.0001
	Spearman’s *ρ*		*p-*value	Spearman’s *ρ*		*p-*value
Gender	0.16		<0.0001	−0.10		0.002
Education	−0.04		0.26	−0.10		0.002
Current employment status	−0.00004		0.99	−0.12		0.0001

AARC: awareness of age-related change; CI: confidence interval.

## The relationship between pain and AARC gains and losses

Table 4 reports the crude (unadjusted) and adjusted coefficients from the multiple regression exploring the predictive value of pain for perceived AARC gains and perceived AARC losses. Pain was not a significant predictor of participants’ scores on perceived AARC gains in either the unadjusted (*B* = 0.05; 95% CI: −0.03 to 0.12; *p*-value = 0.21) or adjusted (*B* = 0.12; 95% CI: 0.03 to 0.20; *p*-value = 0.25) models.

**Table 4.** Regression with pain as the predictor and AARC gains or losses as the outcome: unadjusted and adjusted unstandardised regression coefficients.

	Variable	*B*	SE	95% CI	*p*-value	β	95% CI	Partial *R*^2^	Total *R*^2^
*AARC-10 SF gains*									
Model 1: Unadjusted	Pain	0.05	0.04	−0.03 to 0.12	0.21	0.04	−0.02 to 0.10		0.01%
Model 2: Adjusted	Pain	0.04	0.04	−0.03 to 0.12	0.25	0.04	−0.03 to 0.10	0.01%	0.3%
	Age	−0.02	0.02	−0.06 to 0.02	0.34	−0.04	−0.11 to 0.04	0.01%	
	Gender	1.57	0.32	0.93 to 2.20	<0.0001	0.15	0.09 to 0.22	0.2%	
	Education	−0.50	0.28	−1.05 to 0.04	0.07	−0.06	−0.12 to 0.005	0.03%	
	Current employment status	−0.35	0.29	−0.92 to 0.23	0.24	−0.05	−0.12 to 0.03	0.01%	
*AARC-10 SF losses*									
Model 1: Unadjusted	Pain	0.36	0.03	0.29 to 0.42	<0.0001	0.33	0.28 to 0.39		11%
Model 2: Adjusted	Pain	0.34	0.3	0.27 to 0.40	<0.0001	0.32	0.26 to 0.37	10%	16%
	Age	0.10	0.2	0.06 to 0.13	<0.0001	0.20	0.13 to 0.27	0.3%	
	Gender	−0.53	0.27	−1.06 to 0.01	0.05	−0.06	−0.12 to 0.00	0.03%	
	Education	−0.67	0.23	−1.13 to −0.21	0.004	−0.08	−0.14 to −0.03	0.1%	
	Current employment status	0.25	0.25	−0.23 to 0.73	0.31	0.04	0.50 to 2.01	0.01%	

AARC: awareness of age-related change; AARC-10 SF gains: scale assessing AARC gains in the five AARC life domains; AARC-10 SF losses: scale assessing AARC losses in the five AARC life domains; *B*: regression coefficient; SE: standard error; β: standardised regression coefficient; CI: confidence interval.

Pain predicted participants’ scores on perceived AARC losses (*B* = 0.36; 95% CI: 0.29 to 0.42; *p*-value < 0.001). The coefficient was similar after adjusting for confounders (*B* = 0.34; 95% CI: 0.27 to 0.40; *p*-value < 0.001). Hence, results show that, while controlling for confounders, a unit increase in participants’ score for pain led to an increase in perceived AARC losses of 0.34. While controlling for confounders, pain uniquely explained 10% of the total amount of variability in perceived AARC losses. The amount of variability in levels of perceived AARC losses explained by pain (10%) is greater than that explained by demographic covariates included in the model (which ranged from 0.1% to 3%); hence, pain is a stronger predictor of perceived AARC losses than age, gender, education level and employment status.

## Discussion

This was the first study exploring the associations of pain with perceived AARC gains and AARC losses in a sample of UK individuals aged 50 and over. We hypothesised that individuals with higher perceived pain would perceive lower AARC gains and higher AARC losses. In line with our hypothesis, we found that after adjusting for confounders higher levels of pain are associated with higher levels of perceived AARC losses. However, contrary to our hypothesis, we found little evidence that perceived pain is associated with higher levels of perceived AARC gains.

The predictive role of pain over perceived AARC losses supports our hypothesis that, as pain limits several aspects of one’s life that are captured with the AARC losses subscale, individuals experiencing pain perceive higher levels of AARC losses. Indeed, the presence of pain may negatively impact on physical,^34^ mental (e.g. increased anxiety and depression)^35–37^ and cognitive health.^15^ Moreover, due to the impact of pain on physical health and emotional well-being, individuals experiencing chronic levels of pain can become unable to work and find it difficult to engage in activities.^35^

The association between more pain and more perceived AARC losses suggests that individuals may interpret pain as a consequence of their increased age; this was expected as older individuals are more likely than younger individuals to experience health issues causing pain.^34,35,38–42^ Even though the experience of pain can be considered as a normative part of ageing, for those individuals whose levels of pain can potentially be decreased (e.g. through engagement in health-promoting behaviours), attributing pain to something out of one’s control, such as ageing, can be maladaptive. Indeed perceived lack of control over levels of pain can result in less engagement in coping strategies, health-related behaviours and social activities,^40,43–45^ as well as in more increased perceptions of pain and more negative emotional well-being.^35,40^

However, individuals can also experience severe levels of pain that negatively impact on several aspects of their lives but cannot be improved through treatment of underlying causes. For these individuals interpreting levels of pain as a consequence of their increased age may be a realistic understanding of the age-related changes that can come with ageing. As both the experience of pain and perceived awareness of negative age-related changes can be related to poor mental health (e.g. depression),^46,47^ individuals experiencing high AARC losses and severe levels of pain – that cannot be treated – may benefit from psychological interventions. Psychological interventions, such as psychotherapy, are indeed effective in reducing the psychological symptoms (e.g. anxiety and depression) that are often associated with the experience of pain. Psychological therapies are also effective in reducing the intensity of perceived pain,^46,48^ especially for those individuals who are more susceptible to the experience of pain. Future psychological interventions aiming to decrease both the perceived AARC losses and the experience of pain could include a component of mindfulness that promotes acceptance of negative age-related changes.^18,47,49–51^ Indeed, a mindful attitude is related to lower experience of AARC losses^49^ and mindfulness programs are effective in reducing levels of pain (e.g. Mindfulness-based stress reduction (MBSR)).^52,53^

The relation between greater levels of pain and more perceived AARC losses is also in line with existing literature reporting the negative associations of perceived AARC losses with self-reported physical health and ability to conduct daily activities.^54^ However, the hypothesised direction of the association between pain and perceived AARC losses could be further investigated with future longitudinal studies. As higher levels of pain explain variability in perceived AARC losses, promoting pain management may be a way of decreasing individuals’ perceptions of age-related limitations. Moreover, older individuals experiencing pain could benefit from interventions that, in addition to teaching strategies to control levels of pain, could promote more positive experiences of ageing.^40,45^ This would be in line with the biopsychosocial model of pain, which assumes that in order to fully understand a person’s response to pain and illness, the interrelationships among biological changes, psychological status and the sociocultural context need to be considered.^35^

The non-significant association that we found between higher levels of pain and higher perceived AARC gains does not support our expectation that individuals with higher levels of pain would perceive lower levels of AARC gains. It may be that the experience of pain makes individuals more likely to be concerned for their health and to engage in behaviours related to pain management; aspects that may be captured with the item assessing gains in the AARC ‘health and physical functioning’ domain (‘With my increased age, I pay more attention to my health’). Moreover, individuals experiencing pain may be more likely to receive and value emotional support, leading to higher scores on the item assessing perceived gains in the AARC ‘interpersonal relations’ domain (‘with my increasing age, I appreciate relationship and people more’). Indeed a recent study showed that higher perceived emotional support is a predictor of more perceived AARC gains.^55^ Finally, existing research shows that whereas individuals experiencing pain are more at risk of experiencing psychopathological symptoms (e.g. depression and anxiety), they are as likely to experience good emotional (e.g. interest in life), psychological (e.g. experiences of growth and becoming a better person) and social (e.g. contribute to society) well-being as people without pain.^56^ A meta-analysis on AARC reports a moderate association between perceived AARC losses and worse physical health,^57^ but a negligible association between AARC gains and physical health.^54^ Hence, existing evidence on AARC, along with the negligible association found between pain and perceived AARC gains in this study, suggests that perceptions of AARC gains may be more influenced by psychosocial variables such as perceived social support,^55^ rather than by individuals’ physical health-related outcomes.

Finally, the regression model exploring whether pain, age, sex, education and current employment explain variability in AARC gains showed that only being a woman is a significant predictor of more perceived AARC gains. This result was unexpected as in the UK validation of the AARC-10 SF age, sex and education were significant predictors of AARC gains, even though age and education explained a small amount of variability in AARC. As this study is based on a subsample of participants (*N* = 1013) taken from the broader sample (*N* = 9410) used in the UK validation of the AARC-10 SF,^24^ it may therefore be that the broader sample size used in the validation of the AARC-10 SF made it possible to detect the small effects of age and education over AARC gains.

The findings of this study need to be interpreted while keeping in mind the following limitations. The sample included a majority of participants who were women, White, unemployed, married, well-educated and who rated their health as being good or excellent; hence, generalisation of results needs to be exercised with caution. Only 22% of participants reported some levels of pain; this is a limitation as chronic pain affects between one third and half of the UK adult population.^6^ A further limitation is the lack of information collected as part of the ‘PROTECT’ study on the presence of chronic health conditions and comorbidity and hence on the causes underlying experienced levels of pain. Finally, it was not possible to distinguish between those participants who experienced transient or chronic levels of pain. As the strength and direction of the associations of pain with perceived AARC gains and losses may differ between individuals experiencing acute levels of pain and individuals experiencing chronic pain, future studies could explore whether the strength of the association between pain and perceived AARC is stronger among individuals living with chronic health conditions involving high levels of pain (such as arthritis).^16,40,58^

Analyses were conducted on data collected through self-report measures; hence, recall bias may be present in participants’ answers.^59^ Moreover, the measure of pain that we used in this study assesses perceptions of the degree to which pain interferes with day-to-day activities, which may or may not correspond to pain intensity.^60^ As perceptions of AARC are formed through individuals’ experience of daily performance and functioning (e.g. noticing the reduced capability or increased effort in doing household chores), perceptions of AARC losses may be most strongly associated with measures capturing the degree to which pain interferes with day-to-day activities rather than to measures assessing pain intensity. Future studies could explore whether the strength of the associations between perceived AARC and pain vary when assessing pain intensity.

Another limitation of this study is the use of a cross-sectional design. Future research could explore the longitudinal relationship between pain and AARC, as pain and self-perceptions of physical health can impact on each other over time.^61^ Moreover, as on average participants reported mild symptoms of depression and anxiety, it may be that symptoms of depression and anxiety mediate the association of pain with AARC. However, due to the lack of longitudinal data, we were unable to test this. Finally, as research shows that there can be day-to-day fluctuation in levels of pain^62^ and of AARC,^33^ micro-longitudinal studies could explore whether day-to-day fluctuation in pain relates to fluctuation in levels of AARC. Despite the above limitations, this study has methodological strengths including the large sample size and the wide age range of participants, and analyses controlled for potential confounders in the association of pain with AARC gains and losses.

## Conclusion

Overall, results from this study suggest that individuals experiencing pain may be at risk of perceiving higher levels of negative age-related changes due to the negative outcomes often associated with pain. The efficacy of interventions promoting adaptation to the experience of pain may be enhanced if perceptions of age-related changes are targeted.^17^ Indeed, an increase in AARC gains may motivate individuals to engage in those behaviours that help to cope with the experience of pain and to limit the negative health outcomes often associated with high levels of pain.^17,63^ Those individuals experiencing extensive functional limitations due to pain may benefit from interventions promoting acceptance of negative age-related changes, including the experience of pain.^18,47,49–51^ Whether the associations we found are consistent in samples of individuals experiencing higher levels of chronic pain remains to be explored. As the experience of pain is not significantly associated with perceived AARC gains, perceived AARC gains may not be informative of a better physical state or healthier ageing. Future research is needed to better understand why individuals with more pain perceive higher levels of AARC losses. The associations between pain and perceived AARC gains and losses and potential mediators need to be further explored with longitudinal studies.
